# Prognostic value of skeletal muscle mass during tyrosine kinase inhibitor (TKI) therapy in cancer patients: a systematic review and meta-analysis

**DOI:** 10.1007/s11739-020-02589-5

**Published:** 2020-12-18

**Authors:** Emanuele Rinninella, Marco Cintoni, Pauline Raoul, Francesca Romana Ponziani, Maurizio Pompili, Carmelo Pozzo, Antonia Strippoli, Emilio Bria, Giampaolo Tortora, Antonio Gasbarrini, Maria Cristina Mele

**Affiliations:** 1 UOC di Nutrizione Clinica, Dipartimento di Scienze Mediche e Chirurgiche, Fondazione Policlinico Universitario A. Gemelli IRCCS, Largo A. Gemelli 8, 00168 Rome, Italy; 2 Scuola di Specializzazione in Scienza Dell’Alimentazione, Università di Roma Tor Vergata, Via Montpellier 1, 00133 Rome, Italy; 3 UOSD di Nutrizione Avanzata in Oncologia, Dipartimento di Scienze Mediche e Chirurgiche, Fondazione Policlinico Universitario A. Gemelli IRCCS, Largo A. Gemelli 8, 00168 Rome, Italy; 4 UOC di Medicina Interna e Gastroenterologia, Dipartimento di Scienze Mediche e Chirurgiche, Fondazione Policlinico Universitario A. Gemelli IRCCS, Largo A. Gemelli 8, 00168 Rome, Italy; 5 Dipartimento di Medicina e Chirurgia Traslazionale, Università Cattolica Del Sacro Cuore, Largo F. Vito 1, 00168 Rome, Italy; 6 Comprehensive Cancer Center, Fondazione Policlinico Universitario A. Gemelli IRCCS, Largo A. Gemelli 8, 00168 Rome, Italy

**Keywords:** Skeletal muscle mass, Tyrosine kinase inhibitors, Chemotherapy toxicity, L3 skeletal muscle index, Survival, Personalized medicine

## Abstract

Low muscle mass has been associated with worse clinical outcomes in various cancers. This work investigated whether, during tyrosine kinases inhibitors (TKIs) therapy, low muscle mass was associated with treatment toxicity and survival outcomes. A systematic literature search was performed in Pubmed, Web of Science, and Scopus databases from inception to June 2020, based on fixed inclusion and exclusion criteria. Effect sizes were estimated with hazard ratios (HR) and odds ratios (OR) with 95% confidence interval (CI) and heterogeneity was assessed by measuring inconsistency (*I*^2^) based on the Chi squared test. A total of 24 retrospective studies were identified, enrolling patients treated with sorafenib (*n* = 12), sunitinib (*n* = 6), lenvatinib (*n* = 3), regorafenib (*n* = 2), gefitinib (*n* = 1), imatinib (*n* = 1), and pazopanib (*n* = 1). Thirteen studies were deemed eligible for pooled analyses. Meta-analyses found a significant effect of low muscle mass on dose-limiting toxicity (DLT) (OR 2.40, 95% CI 1.26–4.58, *p* = 0.008, *I*^2^ = 51%) in patients treated with TKI therapy. A subgroup analysis by treatment showed an association between DLT and low muscle during sorafenib or sunitinib, although not significant. A significant association between low skeletal muscle index and poorer overall survival was observed in HCC patients treated with sorafenib (HR 1.45, 95% CI 1.07–1.96, *p* = 0.02). For other TKIs, although some results showed an association between low muscle mass and worse outcomes, the number of studies for each TKI therapy was too small to reach conclusions. Skeletal muscle mass could influence the prognosis of some TKI-treated patients. This effect is demonstrated in sorafenib-treated HCC patients but remains almost unexplored in other cancer patients undergoing TKI therapy. Further prospective studies with large sample size and sufficient follow-up are needed to clarify the role of muscle mass in the metabolism of TKI-based cancer treatment, and its association with toxicity and survival.

**Supplementary Information:**

The online version contains supplementary material available at 10.1007/s11739-020-02589-5.

## Introduction

Muscle wasting represents the primary nutritional issue observed in cancer patients. Loss of skeletal muscle is present in over 50% of newly diagnosed oncologic patients [1, 2] and is related to the catabolic effects of cancer-induced inflammation, such as decreased protein synthesis, increased muscle proteolysis, and hypermetabolism [3]. Furthermore, during antineoplastic treatments, the loss of lean body mass is exacerbated by common treatment side effects such as nausea and loss of appetite, which reduce patient intake of calories and proteins. Low muscle mass has been recognized as a prognostic factor of morbidity and mortality in various types of malignancies such as lung [4], pancreatic [5], gastric [6], hepatic [7], renal [8], and colorectal [9] cancers. Several methods and instruments determine the quantity and quality of muscle mass such as dual-energy X-ray absorptiometry [10], bioelectrical impedance analysis [11], magnetic resonance imaging, and computed tomography (CT) [12]. CT scan imaging, routinely used at diagnosis for tumor staging, is to-date considered the gold standard non-invasive tools to assess muscle quantity and quality [12]. Thus, in recent years, the assessment of muscle wasting has become of great interest in oncology.

Tyrosine kinase inhibitors (TKIs) are effective agents in a wide range of tumor types, including lung cancer [13], colorectal cancer [14], gastrointestinal stromal tumors (GISTs) [15], renal cell carcinoma (RCC) [16], and hepatocellular carcinoma (HCC) [17, 18]. TKIs target enzymes that are responsible for the activation of several intracellular molecular pathways often involved in tumor cell proliferation, such as phosphatidylinositol 3-kinase (PI3K), thymoma viral proto-oncogene (AKT), and mammalian target of rapamycin (mTOR) [19–21]. PI3K-AKT-mTOR pathway plays a key role in muscle protein synthesis. Indeed, the activation of the AKT/mTOR pathway and its downstream targets is essential for regulating skeletal muscle fiber size [20]. Given that the PI3K-AKT-mTOR pathway plays a key role in muscle protein synthesis [19], muscle wasting during TKI therapy has become an important clinical concern. Thus, a growing number of studies [22–28] evaluated the impact of different TKIs on muscle mass changes during treatment in cancer patients. Muscle wasting during TKI therapy may worsen treatment toxicities, such as diarrhea, hand–foot syndrome, rash, and fatigue, limiting the patient’s ability to receive full-dose treatment and resulting in dose reductions and early treatment termination. In patients with low muscle mass, overdose-like effects may occur and lead to dose-limiting toxicity (DLT) [29], defined as any toxicity leading to dose reduction, temporary treatment discontinuation, or permanent treatment discontinuation [30]. Several retrospective studies assessed the relationship between muscle mass and treatment outcomes. This systematic review and meta-analysis aims to evaluate the association between muscle mass quantity or quality and toxicities, overall survival (OS), and progression-free survival (PFS), in patients undergoing TKI therapy.

## Materials and methods

This systematic review was performed according to the Cochrane Handbook for systematic reviews [31] and the preferred reporting items for systematic reviews and meta-analyses (PRISMA) statement [32].

### Eligibility criteria

We included studies that met all of the following criteria:

- Studies with prospective or retrospective designs.
- Studies enrolling adult patients diagnosed with cancer and exclusively undergoing any type of TKI therapy (afatinib, alectinib, axitinib, bosutinib, brigatinib, cabozantinib, ceritinib, crizotinib, dasatinib, erlotinib, gefitinib, ibrutinib, imatinib, lapatinib, lenvatinib, nilotinib, osimertinib, pazopanib, ponatinib, regorafenib, ruxolitinib, sorafenib, sunitinib or vandetanib).
- Studies measuring muscle-mass quantity or quality. Included studies could report lumbar skeletal muscle index (SMI; cm^2^/m^2^), skeletal muscle mass (SMM; cm^2^/m^2^), skeletal mass area (SMA; cm^2^), psoas index (PI; cm^2^/m^2^), skeletal muscle mass density (SMD; Hounsfield units (HU)).

### Outcomes

The outcomes of interest are as follows:

- toxicity endpoints such as DLT, rate of treatment discontinuation due to toxicity, rate of dose reduction due to toxicity, and the total number of adverse events grades ≥ 3 according to the National Cancer Institute (NCI) Common Terminology Criteria for Adverse Events (CTCAE) [33].
- OS defined as the time from the day of the start of TKI treatment to death from any cause.
- PFS defined as the time from the day of TKI treatment to the day on which the first event of disease progression was diagnosed or the day of death from any cause.

### Search strategy and study selection

A systematic literature search was performed from the inception of the electronic databases (MEDLINE, via PubMed, the Institute for Scientific Information Web of Science and Scopus) on 25th June 2020. The search string for each database is described in Appendix 1 (Supplementary data). The search strategy was limited to English language articles. Also, a cross-reference search of eligible articles was performed to check studies that were not found during the computerized search. All articles generated from the electronic search were imported into Mendeley© (Elsevier, Amsterdam, The Netherlands), a reference management software, and duplicates were removed. Two researchers (E.R. and P.R.) independently screened abstracts and titles, then read the full text of relevant studies. Any disagreements were resolved by consensus or by a third reviewer (M.C.M.).

### Data extraction and reporting

Information was collected using an Excel© (Microsoft Office, USA) spreadsheet specifically developed for this study. Each full-text article was retrieved, and any ineligible articles were excluded from the reasoning reported. Differences in judgment between two reviewers (P.R.; E.R.) were settled by discussion and consensus. Data extraction included the first author’s name, year of publication, study design, relevant objectives, patient characteristics, sample size, method of muscle mass assessment, time of muscle-mass assessment, outcomes, and results.

### Statistical analysis

Meta-analyses were performed to estimate the effect of muscle mass on DLT and OS in cancer patients undergoing TKI therapy. Included studies defined low skeletal muscle mass using a cut-off value of muscle area (with or without normalization for sex and/or height). Outcomes were compared between patients with low muscle mass and those without low skeletal muscle mass (high muscle mass). For DLT, an analysis was performed by extracting the odds ratio (ORs) with corresponding 95% confidence intervals (CIs) from primary studies. For survival data, analyses were performed by extracting hazard ratio (HR) with 95% CI from primary studies. For OS, two separate analyses were performed—one included unadjusted HRs (extracted from univariate analysis), and one included adjusted HRs (extracted from multivariate analysis) from each study. A *p* value of < 0.05 was considered statistically significant. Study heterogeneity was evaluated via Cochrane’s *Q* test and the *I*^2^ statistic. The *I*^2^ value of 25%, 50%, and 75% as cut-off points represented low, moderate, and high degrees of heterogeneity, based on Cochrane’s rough guide to the interpretation of *I*^2^ [34]. A Cochrane’s *Q* statistical *P* value < 0.10 and/or *I*^2^ > 50% were deemed as statistically significant heterogeneity. Given the anticipated variability in the cut-off threshold to define low skeletal muscle mass and the clinical characteristics among the included studies, summary estimates were calculated using the random-effects models of DerSimonian and Laird. When possible, subgroup analyses were performed to explore sources of heterogeneity such as cancer type and treatment. All statistical analyses were performed using Review Manager software from the Cochrane Collaboration (RevMan Version 5.3. Copenhagen: The Nordic Cochrane Centre).

### Quality assessment

In this work, all included cohort studies are retrospective. We evaluated all studies with the Newcastle–Ottawa Scale (NOS) representing a valid tool assessing the quality of non-randomized studies in meta-analyses, including cohort studies (retrospective and prospective) [35]. The NOS criteria associated with the selection of the cohort were as follows: representativeness of the exposed cohort; selection of the non-exposed cohort; ascertainment of exposure; and demonstration that an outcome of interest was not present at the beginning of the study. Then, NOS criteria involving the comparability of the cohorts were as follows: the study controlled age and sex as confounders and additional confounding factors. Finally, the following NOS criteria involved the assessment of the outcome were as follows: independent blind assessment or record linkage, sufficiently long follow-up for outcome to occur; and adequacy of cohort follow-up. The judgement for each NOS criteria involves answering a question, with answers ‘Yes’ indicating a low risk of bias, ‘No’ indicating a high risk of bias, and ‘Unclear’ indicating either lack of information or uncertainty over the potential for bias. Two investigators independently assessed the methodological quality of the included cohort studies using NOS criteria. Differences in judgment among reviewers were settled by discussion and consensus.

## Results

### Study selection

**Fig. 1:**
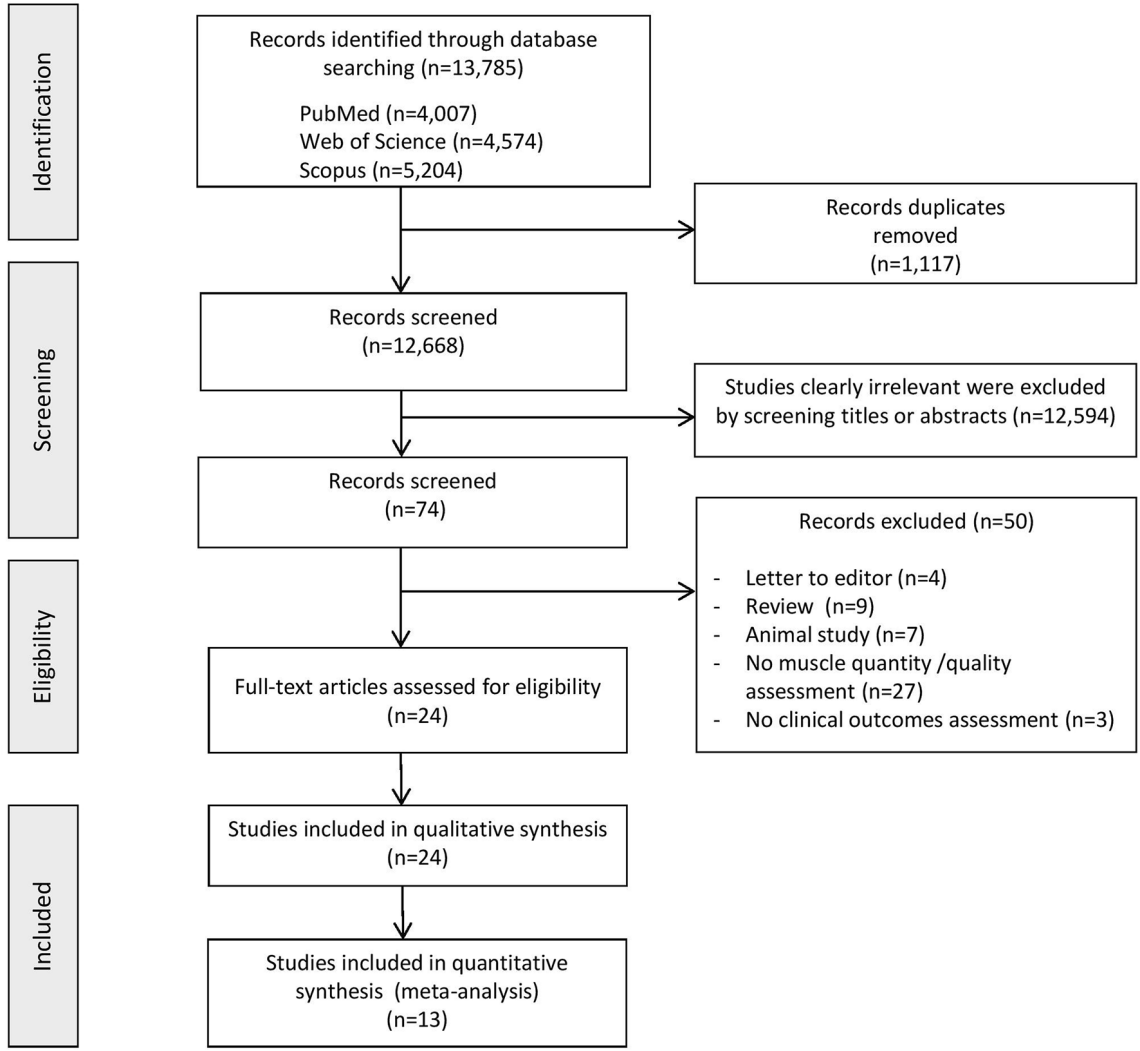
Preferred reporting items for systematic reviews and meta-analyses (PRISMA) flow diagram

The flow diagram in Fig. 1 displays the results of the literature search and study selection process. Thirteen thousand seven hundred eighty-five publications, of which duplicates 1,117, were initially identified and 12,594 irrelevant studies were discarded. From 74 screened records, 50 were excluded for the following reasons: letters to the editor (*n* = 4), reviews (*n* = 9), animal studies (*n* = 7), no muscle quantity or quality assessment (*n* = 27), no assessment of clinical outcomes (*n* = 3). Twenty-four [15, 24, 26–28, 36–54] studies were included in the systematic review, of which 13 [24, 26, 39–42, 44–48, 50, 54] in the meta-analyses.

### Study characteristics

Descriptive results of each included studies are reported in Table 1 and Table 2. All included studies were retrospective. Publication date ranged from 2010 [39] to 2020 [51–53] and sample size from 21 [51] to 365 [26] patients. All enrolled patients had an advanced stage cancer. All included studies used CT imaging, performed in the pre-treatment period, to calculate muscle mass. The CT indexes to measure muscle mass varied across studies; twenty-one studies measured SMI, two measured SMA [38, 39], one measured SMM [51], one psoas index PI [43] and one SMD [45]. The muscle mass was quantified in patients undergoing therapy with gefinitib (one study [36]), imatinib (one study [15]), lenvatinib (three studies [22, 52, 53]), pazopanib (one study [37]), regorafenib (two studies [38, 51]), sorafenib (twelve studies [24, 26–28, 39–46]) and sunitinib (six studies [27, 47–50, 54]). Cut-offs values varied across the studies, such as 55.4 cm^2^/m^2^ for men and 38.9 cm^2^/m^2^ for women [39, 40, 47, 48], or 42 cm^2^/m^2^ for men and 38 cm^2^/m^2^ for women [51–53] or 43 cm^2^/m^2^ for men with a body mass index (BMI) < 25 kg/m^2^, 53 cm^2^/m^2^ for men with a BMI > 25 kg/m^2^, and 42 cm^2^/m^2^ for women [15, 44, 54] or median cut-offs [26, 42]. One study [51] measured the SMM decrease ≤ or > 2%.

**Table 1:** Descriptive results of the included studies regarding toxicity (classified by TKI type)

TKI	First Author, Year, Country	Variables	Time of toxicity assessment	Comparison	Cut-offs values (high vs. low muscle)	Adjustment	HR (95% CI) or OR (95% CI) or mean (± SD) or median (IQR) or %	p value
Gefitinib	Rossi, 2018, Italy [36]	Number of all-grade toxicities*, %	From beginning of treatment until disease progression or death from any causes	Low SMI vs. high SMI	55 cm^2^/m^2^ for men 39 cm^2^/m^2^ for women	Univariable	92% vs. 69%	NS
Imatinib	Moryoussef, 2015, France [15]	Number of all-grade toxicities*, mean ± SD	3 months after starting treatment	Low SMI vs. high SMI	43 cm^2^/m^2^ for men with BMI < 25 kg/m^2^ 53 cm^2^/m^2^ for men with BMI ≥ 25 kg/m^2^ 42 cm^2^/m^2^ for women	Univariable	Mean 4.1 ± 1.9 vs. 1.7 ± 1.8	< 0.01
Lenvatinib	Uojima, 2020, Japan [53]	Number of severe toxicities*	In the first two months	Low SMI vs. high SMI	42 cm^2^/m^2^ for men 38 cm^2^/m^2^ for women	Univariable	OR 3.33 (1.27–8.74)	0.015
Regorafenib	Gokyer, 2019, Turkey [38]	DLT	At the end of the 3 cycles of treatment	Low SMI vs. high SMI	49 cm^2^/m^2^ for men 31 cm^2^/m^2^ for women	Univariable Multivariable	HR 15.6 (1.72–140.82) HR 12.99 (1.25–134.80)	0.01 0.03
Sorafenib Sunitinib Others	Gu, 2017, China [27]	DLT, %	During the first 3 months’ treatment	Muscle loss ≥ 5% vs. muscle loss < 5%	NA	Univariable	40% vs. 21.7%	NS
Sorafenib	Naganuma, 2017, Japan [42]	Discontinuation of treatment due to toxicity	NA	Low SMI vs. high SMI	Median L3-SMI 43 cm^2^/m^2^ for men 36 cm^2^/m^2^ for women	Univariable	HR 1.610 (0.516–5.019)	NS
Sorafenib	Labeur, 2019, Netherlands [45]	Number of adverse events*	From start of sorafenib until disease progression	Low SMI vs.. high SMI Low SMD vs. high SMD	NR	Univariable Univariable	NA NA	NS NS
Sorafenib	Mir, 2012, France [40]	DLT, %	During the first month of treatment	Low SMI vs. high SMI	55.4 cm^2^/m^2^ for men 38.9 cm^2^/m^2^ for women	Univariable	81.8% vs. 31.0%	0.005
Sorafenib	Huillard, 2019, France [26]	DLT, %	Within 6 months from the initiation of therapy	Low SMI vs. high SMI	Median SMI	Univariable	53.3% vs. 44.7%	NS
Sorafenib	Sawada, 2019, Japan [46]	Discontinuation rate of treatment due to toxicity	During sorafenib treatment (138 days)	Low SMI vs. high SMI	36.2 cm^2^/m^2^ for men 29.6 cm^2^/m^2^ for women	Univariable Multivariable	HR 3.185 (1.713–5.922) HR 3.396 (1.731–6.664)	< 0.001 < 0.001
Sorafenib	Hiraoka, 2017, Japan [43]	Discontinuation of sorafenib due to toxicity, %	3 and 6 months after starting of treatment	Low PI vs. high PI	4.24 cm^2^/m^2^ for men 2.50 cm^2^/m^2^ for women	Univariable	55.1% vs. 37.0%	NS
Sorafenib	Antonelli, 2018, Italy [44]	Total number of adverse events grade ≥ 3 *	During the first cycle of treatment	Low SMI vs. high SMI	43 cm^2^/m^2^ for men with BMI < 25 kg/m^2^ 53 cm^2^/m^2^ for men with BMI > 25 kg/m^2^ 42 cm^2^/m^2^ for women	Univariable	62 vs. 40	0.04
Sorafenib	Antoun, 2010, France [39]	DLT, %	NA	Low SMI vs. high SMI	55.4 cm^2^/m^2^ for men 38.9 cm^2^/m^2^ for women	Univariable	37% vs. 5.5%	0.02
Sorafenib	Imai, 2015, Japan [41]	Dose reduction due to toxicity	NA	Low SMI vs. high SMI	NR	Univariable	HR 0.979 (0.928–1.032)	NS
		Discontinuation of treatment due to toxicity	NA			Univariable	HR 0.951 (0.891–1.015)	NS
Sunitinib	Huillard, 2013, France [47]	DLT, %	During the first cycle of treatment	Low SMI and BMI < 25 kg/m^2^ vs. high SMI or BMI > 25 kg/m^2^	55.4 cm^2^/m^2^ for men	Univariable	50% vs. 19.5%	0.01
					38.9 cm^2^/m^2^ for women			
Sunitinib	Ishihara, 2018, Japan [50]	DLT, %	NA	ΔSMI < 0 and those with ΔSMI ≥ 0	NA	Univariable	57.9% vs. 54.8%	NS
		Discontinuation of treatment due to toxicity, %				Univariable	39.5% vs. 16.1%	0.0163
Sunitinib	Cushen, 2014, Ireland [48]	DLT, %	After 4 cycles of treatment (6 months)	SMI, Q1 (< 44.8 cm^2^/m^2^) vs. Q4 (> 63.2 cm^2^/m^2^)	55.4 cm^2^/m^2^ for men	Univariable	92% vs. 57%	0.05
				Low SMI vs. high SMI	38.9 cm^2^/m^2^ for women	Univariable	77.7% vs. 70%	NS
Sunitinib	Narjoz, 2015, France [49]	Any grade ≥ 3 toxicity*	During the first cycle of treatment	LBM (kg) as continuous variable	NA (continuous variable)	Multivariable	NA	NS
Sunitinib	Ishihara, 2016, Japan [54]	DLT, %	During the first cycle	Low SMI vs. high SMI	43 cm^2^/m^2^ for men with BMI < 25 kg/m^2^ 53 cm^2^/m^2^ for men with BMI > 25 kg/m^2^ 42 cm^2^/m^2^ for women	Univariable	51.1% vs. 50.0%	NS
Pazopanib Sunitinib	Köstek, 2019, Turkey [37]	DLT	During the first cycle	Baseline SMA	NA	Univariable	NR	NS

* according to the NCI-CTC; *CI* confidence interval; *DLT* dose-limiting toxicity; *HR* hazard ratio; *HU* Hounsfield units; *LBM* lean body mass; *NA* not applicable; *NR* not reported; *NS* non-significant; *OR* odd ratio; *PI* psoas index; *Q* quartile; *SD* standard deviation; *SMA* skeletal muscle area (cm^2^); *SMD* skeletal muscle density; *SMI* skeletal muscle index (cm^2^/m^2^); *SMM* skeletal muscle mass; *TKI* tyrosine kinase inhibitor; *vs.*, versus

**Table 2:** Descriptive results of the included studies regarding survival outcomes (classified by TKI type)

TKI	First author, year, country	Outcomes	Comparison	Cut-off values (high vs. low muscle)	Adjustment	HR (95% CI) or OR (95% CI) or mean or median or %	p value
Gefitinib	Rossi, 2018, Italy [36]	OS	High SMI vs. low SMI	55 cm^2^/m^2^ for men 39 cm^2^/m^2^ for women	Univariable	Median:12.6 (4.7–16.1) vs. 23.5 (15–33.3) HR: 0.45 (0.22–0.96)	0.035
Lenvatinib	Uojima, 2020, Japan [53]	OS	Low SMI vs. high SMI	42 cm^2^/m^2^ for men 38 cm^2^/m^2^ for women	Univariable Multivariable	OR 2.22 (1.11–4.45) OR 2.25 (1.09–4.62)	0.025 0.028
Lenvatinib	Yamazaki, 2020, Japan [52]	PFS	Low SMI vs. high SMI	42 cm^2^/m^2^ for men 38 cm^2^/m^2^ for women	Univariable Multivariable	NR HR 2.488 (1.058–5.846)	0.017 0.037
Regorafenib	Gokyer, 2019, Turkey [38]	OS PFS	Low SMI vs. high SMI	49 cm^2^/m^2^ for men 31 cm^2^/m^2^ for women	Univariable Univariable	NR NR	NS NS
Regorafenib	Bekir, 2020,Turkey [51]	OS	SMM decrease ≥ 2% vs. SMM decrease < 2%	NA	Univariable	HR 2.82 (1.07–7.42)	0.03
Sorafenib Sunitinib Others	Gu, 2017, China [27]	OS PFS	Muscle loss ≥ 5% vs. muscle loss < 5%	NA	Univariable Univariable	HR 2.186 (1.209–3.952) HR 1.745 (1.102–2.762)	0.010 0.018
Sorafenib	Naganuma, 2017, Japan [42]	OS	Low SMI vs. high SMI	Median L3-SMI 43 cm^2^/m^2^ for men 36 cm^2^/m^2^ for women	Univariable Univariable Multivariable Multivariable	Male: HR 1.916 (1.008–3.642) Female: HR 1.279 (0.404–4.045) Male: HR 2.315 (1.125–4.765) Female: HR 1.835 (0.372–9.040)	0.047 NS 0.023 NS
Sorafenib	Labeur, 2019, Netherlands [45]	OS OS	Low SMI vs. high SMI Low SMD vs. high SMD	NR	Univariable Univariable	HR 1.20 (0.94–1.54) HR 0.97 (0.75–1.24)	NS NS
Sorafenib	Mir, 2012, France [40]	OS PFS	Low SMI vs. high SMI	55.4 cm^2^/m^2^ for men 38.9 cm^2^/m^2^ for women	Univariable Univariable	Median: 7.4 (1.9–19.3) vs. 11.0 (7.7–16.5) Median: 2.5 (1.3–16.1 vs. 4.6 (2.5–7.7)	NS NS
Sorafenib	Saeki, 2019, Japan [24]	OS	% SMI changes from baseline to 3 months after treatment	NA	Univariable Multivariable	HR 0.506 (0.300–0.864) HR 0.55 (0.317–0.983)	0.013 0.044
Sorafenib	Sawada, 2019, Japan [46]	OS PFS	Low SMI vs. high SMI	36.2 cm^2^/m^2^ for men 29.6 cm^2^/m^2^ for women	Univariable Multivariable Univariable Multivariable	HR 2.629 (1.341–5.154) HR 1.153 (0.538–2.474) HR 1.899 (1.029–3.506) HR 1.233 (0.653–2.327)	0.004 NS 0.04 NS
Sorafenib	Hiraoka, 2017, Japan [43]	OS	Low PI vs. high PI	4.24 cm^2^/m^2^ for men 2.50 cm^2^/m^2^ for women	Univariable	Median: 7.6 vs. 15.6	0.042
Sorafenib	Antonelli, 2018, Italy [44]	OS	Low SMI vs. high SMI	43 cm^2^/m^2^ for men with BMI < 25 kg/m^2^ 53 cm^2^/m^2^ for men with BMI > 25 kg/m^2^ 42 cm^2^/m^2^ for women	Univariable Multivariable	HR 1.71 (1.12–2.71) HR 1.63 (1.05–2.53)	0.01 0.03
Sorafenib	Imai, 2015, Japan [41]	OS	Low SMI vs. high SMI	NR	Univariable Multivariable	HR 0.904 (0.830–0.984) HR 0.909 (0.836–0.985)	0.02 0.02
Sorafenib Lenvatinib	Uchikawa, 2019, Japan [28]	OS	ΔSMI < 0 and those with ΔSMI ≥ 0 ΔSMI: (post SMI – pre SMI) from initiation to evaluation	NA	Univariable	NR	NS
Sunitinib	Huillard, 2013, France [47]	OS PFS	Low SMI and BMI < 25 kg/m^2^ vs. high SMI and BMI > 25 kg/m^2^	55.4 cm^2^/m^2^ for men 38.9 cm^2^/m^2^ for women	Univariable Univariable	NR NR	NS NS
Sunitinib	Ishihara, 2018, Japan [50]	OS	ΔSMI < 0 and those with ΔSMI ≥ 0	L3 SMI (cm^2^/m^2^) calculated from CT scan ΔSMI (relative SMI change during the initial two cycles of treatment)	Univariable Multivariable	HR 4.08 (1.96–9.32) HR 4.53 (2.15–10.5)	0.0001 < 0.0001
Sunitinib	Cushen, 2014, Ireland [48]	OS PFS	SMI, Q1 (< 44.8 cm^2^/m^2^) vs. Q4 (> 63.2 cm^2^/m^2^) Low SMI vs. high SMI	55.4 cm^2^/m^2^ for men 38.9 cm^2^/m^2^ for women	Univariable Univariable	NR NR	NS NS
Sunitinib	Ishihara, 2016, Japan [54]	OS PFS	Low SMI vs. high SMI	43 cm^2^/m^2^ for men with BMI < 25 kg/m^2^ 53 cm^2^/m^2^ for men with BMI > 25 kg/m^2^ 42 cm^2^/m^2^ for women	Univariable Multivariable Univariable Multivariable	HR 4.29 (1.72–13.0) HR 2.29 (0.73–8.16) HR 3.15 (1.66–6.41) HR 2.54 (1.19–5.65)	0.0012 0.0004 < 0.0001 0.02
Pazopanib Sunitinib	Köstek, 2019, Turkey [37]	OS PFS	Baseline SMA	NA	Univariable Univariable	NR NR	NS NS

*BMI* body mass index; *CI* confidence interval; *HR* hazard ratio; *HU* Hounsfield units; *LBM* lean body mass; *NR* not reported; *NS* non-significant; *OR* odds ratio; *OS* overall survival; *PFS* progression-free survival; *PI* psoas index; *Q* quartile; *SD* standard deviation; *SMA* skeletal muscle area (cm^2^); *SMD* skeletal muscle density; *SMI* skeletal muscle index (cm^2^/m^2^); *SMM* skeletal muscle mass; *TKI* tyrosine kinase inhibitor; vs., versus

Regarding endpoints, 9 out of 20 studies assessed DLT [26, 27, 38–40, 47, 48, 50, 54], five reported treatment discontinuations due to toxicity [41–43, 46, 50], four measured toxicity as the total number of adverse events [36, 44, 45, 49], the number of severe adverse events [53] or the rate of dose reduction [41]. Treatment toxicity was evaluated during the first cycle of treatment [44, 47, 49, 54], at three [15, 27, 43], or six months [26, 43, 48] after starting treatment. Moreover, OS was assessed in 20 studies while PFS was assessed in eight studies [37, 38, 40, 47, 49, 50, 52, 54].

### Quality assessment

The quality assessment of each NOS criterion for each study was detailed in Appendix 2 (Supplementary data).

Twenty-three studies out of the total were truly representative of population-based study, except for Antoun et al. [39] who selected only male patients. All studies selected the non-exposed cohort from the same community as the exposed cohort, except for Gu et al. [27] where there was not a selected non-exposed cohort. As regards ascertainment of exposure, all studies directly measured muscle mass and previously defined specific cut-offs values. In all studies, outcomes of interest were not present at the start of the study. Twenty-two studies out of the total controlled for confounders such as sex, age, and other additional confounding variables such as cancer stage, number of metastatic sites, time from diagnosis to treatment, and comorbidities. For only two studies [47, 48], the confounders that were adjusted for were not clearly stated. For all studies, imaging analyses were performed by investigators/radiologists who were blinded to the patient outcomes. As regards follow-up, four studies [40, 47, 49, 54] did not have adequate follow-up time to assess DLT, indeed, DLT was assessed during the first cycle of TKI treatment and two studies [41, 42] did not specify the follow-up time. Finally, overall, studies had an adequate follow-up of cohorts whereas for four studies the number of patients lost to follow up was unclear [41, 42, 48, 54].

### Results

The results of the studies are reported in Table 1 for treatment toxicity and Table 2 for survival outcomes (OS and PFS).

#### Sorafenib

**Fig. 2:**
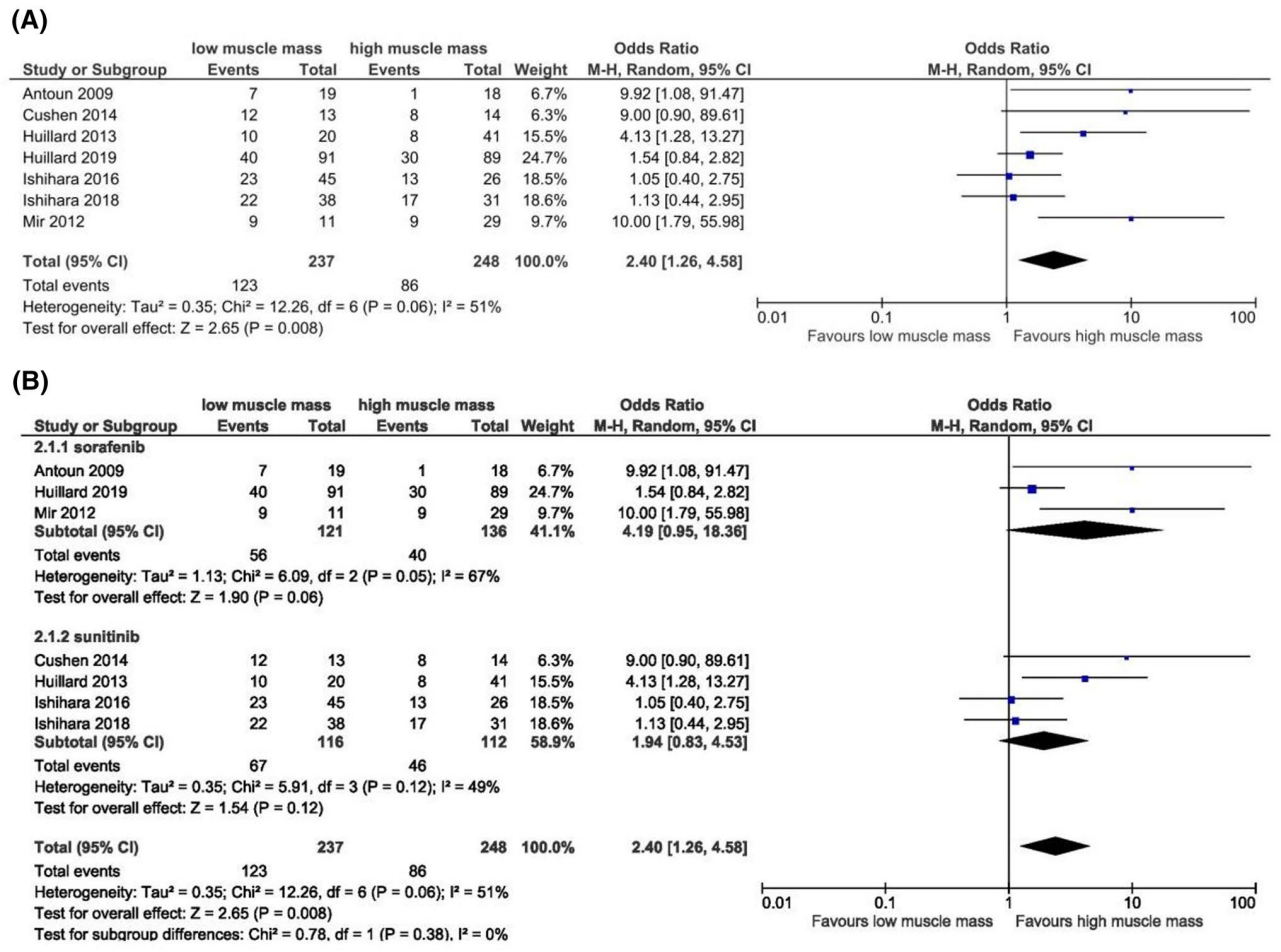
**a** Forest plot evaluating the effect of muscle mass on DLT due to TKI treatment (sorafenib, sunitinib). **b** Subgroup analysis by TKI type. *CI* confidence interval; *DLT* dose-limiting toxicity; *IV* inverse variance

Sorafenib is approved for the treatment of advanced HCC, RCC, and unresectable thyroid cancer [55]. Sorafenib blocks receptor tyrosine kinase signaling, such as vascular endothelial growth factor receptor (VEGFR), platelet-derived growth factor receptor (PDGFR), c-KIT, and RET, and inhibits downstream Raf serine/threonine kinase activity to prevent tumor growth by anti-angiogenic, anti-proliferative, and/or pro-apoptotic effects [56]. Regarding toxicity, the results of the different studies [26, 39–46] varied according to muscle mass assessment methods and endpoints (Table 1). Meta-analyses of data were performed to evaluate the effect of muscle mass on DLT during TKI therapy (sorafenib or sunitinib). A total of 485 patients from seven studies were included in the analysis of ORs. Compared to patients with low muscle mass, patients with normal/high muscle mass reported a significantly lower DLT (OR 2.40, 95% CI 1.26–4.58, *p* = 0.008) with moderate significant heterogeneity (*I*^2^ = 51%, *P* > 0.05) (Fig. 2a). However, a subgroup analysis (Fig. 2b) by treatment showed that DLT during sorafenib treatment was not associated with muscle mass (OR 4.19, 95% CI 0.95–18.36, *p* > 0.05). The significant heterogeneity (*I*^2^ = 67%, *P* > 0.10) is probably due to cancer type. Indeed, this meta-analysis included only three studies from thyroid and HCC patients. Further studies with large and homogeneous sample size are required to confirm the significant effect of muscle mass on DLT in sorafenib-treated HCC patients, demonstrated in two studies [39, 40].

**Fig. 3:**
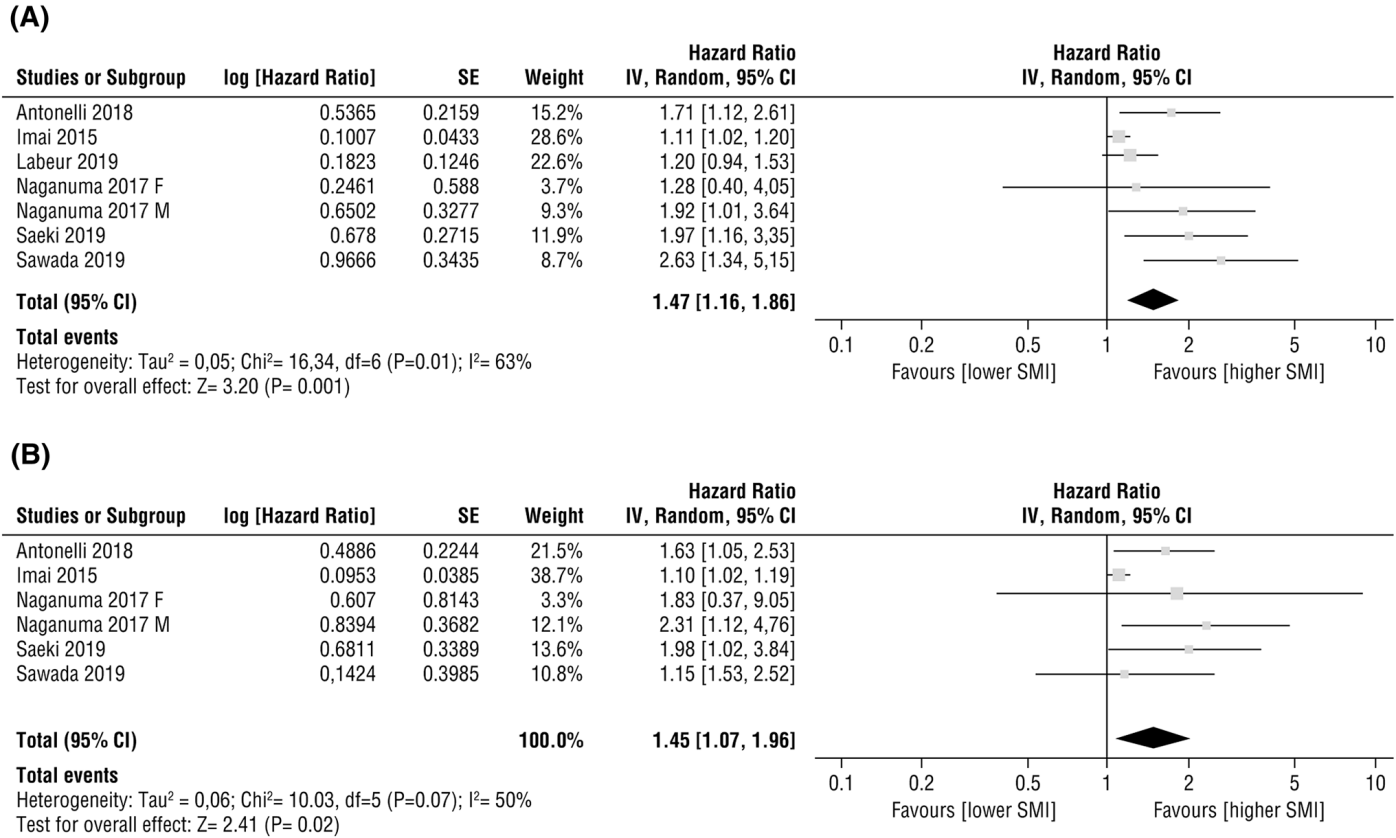
Forest plots evaluating the effect of SMI on overall survival in advanced HCC patients undergoing sorafenib treatment (**a**) HR from univariate analysis (**b**) HR from multivariate analysis. *CI* confidence interval; *HCC* hepatocellular carcinoma; *HR* hazard ratio; *IV* inverse variance; *SMI* skeletal muscle index

Meta-analyses assessing the effect of baseline SMI on OS have been performed. A total of 420 patients from six studies [24, 41, 42, 44–46] were included in the analysis of HRs for OS by univariate analysis. Compared to patients with low SMI, patients with normal/high SMI reported a significantly better OS (HR 1.47, 95% CI 1.16–1.86, *p* = 0.001; Fig. 3a). The test for heterogeneity was significant (*P* = 0.01; *I*^2^ = 63%) and a random-effects model was used. Multivariate analyses in five studies [24, 41, 42, 44, 46] were performed to evaluate the effects of confounding factors such as age, gender, body mass index, Child–Pugh score, clinical disease’s stage, and initial dose of sorafenib treatment. A meta-analysis of these adjusted HRs was performed and confirmed that a high SMI at baseline was independently associated with better OS (HR 1.45, 95% CI 1.07–1.96, *p* = 0.02, Fig. 3b). A random-effects model was used; the test for heterogeneity was high (*P* = 0.07; *I*^2^ = 50%).

#### Sunitinib

Sunitinib is a multitargeted TKI mainly acting on VEGFR and PDGFR [16]. Sunitinib is the reference standard of care for first-line treatment of metastatic RCC or pancreatic neuroendocrine tumors and for second-line treatment of patients affected by unresectable and/or metastatic GISTs who failed on previous imatinib therapy [57]. Regarding toxicity due to sunitinib treatment, a subgroup analysis of data from four studies [26, 48, 50, 54] including 228 patients was performed to evaluate the effect of muscle mass on DLT in RCC patients (Fig. 2b). Low muscle mass was not associated with DLT (OR 1.94, 95% CI 0.83–4.53, *p* > 0.05). The test for heterogeneity was moderate and significant (*P* > 0.10; *I*^2^ = 49%). Regarding the effect of muscle mass on survival outcomes, three studies [37, 47, 48] observed a non-significant association between muscle mass and OS/PFS, while two other studies [50, 54] showed a significant association between low SMI and poor OS/PFS in univariate (HR 4.08, 95% CI 1.96–9.32, *p* = 0.0001 [50]; HR 4.29, 95% CI 1.72–13.0, *p* = 0.0012 [54]) and multivariate (HR 4.53, 95% CI 2.15–10.5, *p* < 0.0001 [50]; HR 2.29, 95% CI 0.73–8.16, *p* = 0.0004 [54]) analyses. It was not possible to perform a meta-analysis due to a lack of available HRs from the other two studies [47, 48].

#### Lenvatinib

Lenvatinib is an oral TKI targeting VEGFRs 1–3, fibroblast growth factor receptors (FGFRs) 1–4, PDGFR-α, proto-oncogene receptor tyrosine kinase rearranged during transfection (RET), and c-KIT proto-oncogenes. These tyrosine kinase receptors are implicated in tumor angiogenesis as well as tumor growth and progression [18]. It is approved for the treatment of advanced and metastatic differentiated thyroid carcinoma after radioactive iodine failure and as first-line therapy for the treatment of advanced HCC [58]. As regards toxicity, one study [53] evaluated in 100 patients the number of severe adverse advents during the first two months of lenvatinib treatment, showing a significant association (unadjusted OR 2.22, 95% CI 1.11–4.45, *p* = 0.025; adjusted OR 2.25, 95% CI 1.09–4.62, *p* = 0.028). Two studies [52, 53] assessed, in both univariable and multivariable analyses, a significant association between low SMI at diagnosis and OS (adjusted OR 2.25, 95% CI 1.09–4.62, *p* = 0.028) [53] in patients with HCC and PFS in patients with metastatic thyroid cancer (adjusted HR 2.488, 95% CI 1.058–5.846, *p* = 0.037) [52]. However, one study did not show a significant association between OS and muscle depletion during lenvatinib treatment [28].

#### Regorafenib

Regorafenib is an oral multikinase inhibitor of several protein kinases, including kinases involved in tumor angiogenesis (VEGFRs 1–3), oncogenesis (KIT, RET, RAF1, BRAF, and BRAFV600E), and development of the tumor microenvironment (PDGFR and FGFR) [14]. Given the results of a large, international, multicenter, randomized, placebo-controlled, Phase 3 trial (the CORRECT study) [14], regorafenib is currently approved for second- or third-line treatment of metastatic colorectal carcinoma, after the failure of fluoropyrimidine-, oxaliplatin-, and irinotecan-based chemotherapy, and of anti-VEGF and/or anti-targeted therapy. Moreover, regorafenib is effective as second-line therapy for the treatment of advanced HCC after sorafenib failure [59], and in patients affected by GISTs who had progression or intolerance to imatinib or sunitinib [60, 61]. Regarding toxicity, only one study [38] assessed DLT at the end of three cycles of regorafenib treatment and demonstrated that it was significantly higher in patients with lower SMI at baseline (HR 15.6, 95% CI 1.72–140.82, *p* = 0.01). Moreover, this study showed a non-significant association between low SMI and OS/ PFS [38]. However, a recent study [51] showed that SMM decrease ≥ 2% during regorafenib treatment was significantly associated with a worse OS (HR 2.87; 95% CI 1.07–7.42, *p* = 0.03).

#### Imatinib

Imatinib is a kinase inhibitor approved for the treatment of newly diagnosed adult patients with Philadelphia chromosome-positive chronic myeloid leukemia in the chronic phase, in the blast crisis, in the accelerated phase, or chronic phase after failure of interferon-alpha therapy. It is also used in pediatric patients with Ph + CML in chronic phase who are newly diagnosed, or whose disease has recurred after stem-cell transplant, or who are resistant to interferon-alpha therapy. Imatinib can be administered to adult patients with unresectable, recurrent and/or metastatic dermatofibrosarcoma protuberans and in patients with c-kit positive unresectable and/or metastatic malignant GISTs [62]. Imatinib is a 2-phenyl amino pyrimidine derivative that functions as a specific inhibitor of several tyrosine kinase enzymes. Only one study [15] evaluated the associations between toxicity and muscle mass. Pre-treatment low SMI was not associated with grades 3–4 toxicities, but the mean number of all-grade toxicities was significantly higher in patients with low SMI vs patients with high SMI (4.1 vs. 1.7; *p* < 0.01) after 3 months of treatment [15].

#### Gefitinib

Gefitinib was approved for the treatment of patients with metastatic, EGFR mutation-positive NSCLC [63]. One study [36] suggested that gefitinib-treated patients with low SMI had a trend toward higher cutaneous toxicity, even if not statistically significant, and required more dose reductions. In this study, low SMI did not significantly affect the number of all-grade toxicities according to the NCI-CTCAE but a significant association between low SMI and poorer OS was observed (HR 0.45, 95% CI 0.22–0.96, *p* = 0.035) [36].

#### Pazopanib

Pazopanib is a kinase inhibitor indicated for the treatment of patients with advanced RCC or advanced soft tissue sarcoma, having received prior chemotherapy [64]. Pazopanib is a VEGFRs inhibitor with activity against VEGFRs1-3, PDGFR, c-KIT, and FGFR [65]. One study [37] was found, demonstrating that baseline SMA and LBM were significantly associated neither with DLT due to pazopanib treatment, nor with OS and PFS.

## Discussion

We have systematically reviewed the currently published literature to evaluate the effect of muscle mass on treatment toxicity and survival during TKI therapy. The majority of included studies were related to sorafenib and sunitinib while a small number of recent studies evaluated the impact of muscle mass on clinical outcomes during lenvatinib, regorafenib, imatinib, gefitinib, pazopanib therapies [66].

This review highlights a significant association between low muscle mass and toxicity during TKI therapy. Specifically, in two studies analyzing HCC patients, low muscle mass is significantly associated with DLTs during sorafenib treatment. Moreover, in RCC patients, pooled data from sunitinib studies showed that low muscle mass could be also a risk factor of DLT during treatment, although not significant. As regards imatinib, lenvatinib, regorafenib, low muscle mass during treatment could be significantly associated with toxicity while low muscle mass in patients treated with gefitinib and pazopanib did not appear to be significantly associated with toxicity. These results highlight the complexity of the toxicity responses induced by TKI treatment and its association with muscle mass. We can hypothesize that the association of muscle mass with TKI side effects may depend on cancer type. Especially, in HCC, detrimental effects on the nutritional status of the patient and consequently on the tolerability of the treatment are also due to the underlying concurrent disease, the liver cirrhosis [63]. In addition, the association between muscle mass and toxicity could be influenced by the number of inhibited targets (single versus multikinase inhibitors), the strength of target inhibition (affinity to the tyrosine kinase), and the type of inhibited target [67]. In particular, some TKIs—such as sunitinib, sorafenib, lenvatinib, regorafenib—mainly targets VEGFR. Interestingly, VEGF promotes the growth of myogenic fibers and protects the myogenic cells from apoptosis [68]. Thus, by inhibiting targeted receptor tyrosine kinase such as VEGFR, some TKIs could inhibit muscle growth and dysregulate skeletal muscle fiber size [20], exacerbating the decrease of skeletal muscle mass and the increase of toxicity. Furthermore, a recent study suggested that the inhibition of the Akt/mTOR pathway, a key regulator of the muscle protein synthesis, may explain the marked loss of muscle mass in the long-term use of mTOR inhibitors [69]. In this context, since the majority of TKIs are administered at the same dose regardless of body weight, we can conjecture that patients who had low body weight (and/or low muscle mass) would be at higher risk for toxicity. Only for lenvatinib, the recommended dosage depends on the patient’s body weight: 8 mg if < 60 kg and 12 mg if ≥ 60 kg [70]. Interestingly, an exposure–response relationship was observed between lenvatinib withdrawal and body weight, indicating that dose adjustment with optimal body weight cut-off values improves lenvatinib safety in the treatment of HCC patients [71]. We can further hypothesize that dosage adjustments for body weight—and muscle mass—could improve the drug’s tolerability. Further studies are required to find algorithms to determine weight-based doses for each TKI therapy.

As regards OS and PFS, patients treated with sorafenib and sunitinib were mainly studied, given their long commercial release and use in the clinical practice. A meta-analysis showed that low muscle mass was significantly associated with poor OS in patients with advanced HCC treated with sorafenib. Two studies [52, 53] found similar results in HCC patients undergoing lenvatinib therapy. During sorafenib and lenvatinib therapy, as previously described, low muscle mass could be associated with higher toxicity. It is known that low tolerability of chemotherapy leads to decreased survival and higher recurrence/treatment failure rates [43, 72]. Therefore, in HCC patients treated with sorafenib or lenvatinib, adequate nutritional support should be proposed at diagnosis to counteract potential muscle mass wasting and improve prognosis. Furthermore, in RCC, SMI, and SMD were also assessed in patients treated with targeted therapies including sorafenib, everolimus, and sunitinib [73]. Indeed, a controlled trial showed that high SMD improved OS and PFS of these patients [73]. This could be explained by muscle density which is closely related to muscle lipid content [74] that is linked to inflammatory processes. Indeed, myosteatosis, the biological prerogative of low skeletal muscle radiodensity, often occurs in cancer and other inflammatory diseases [75]. Due to the lack of recognizable early symptoms, RCC cancer is frequently diagnosed at an advanced stage. Consequently, a variety of catabolic pro-inflammatory cytokines, such as tumor necrosis factor-α and interleukin-6 are already induced, influencing myosteatosis. These promising results are needed to be confirmed. Further studies are required to define the threshold values for muscle density and assess its association with outcomes in patients treated with TKIs such as sorafenib and sunitinib. As regards other TKIs, few studies showed a significant association between low muscle mass and OS during gefitinib and regorafenib therapies. These results confirmed that lower muscle mass in cancer patients may be associated with worse OS and PFS [76]. We can hypothesize that, during regorafenib and gefitinib therapy, decreased skeletal muscle mass may be exacerbated with the imbalance between proteolysis and muscle metabolism induced by the inhibition of the Akt/mTOR pathway.

Furthermore, although the effects of mTOR inhibitors on muscle mass have yet to be fully elucidated, a recent narrative review based on a systematic literature search [77] highlighted that the loss of skeletal muscle mass may be exacerbated by different TKI treatments—such as axitinib [22], lenvatinib [25], regorafenib [23, 51], sorafenib [26, 28, 39], or sunitinib [27]. In this context, every effort should be made to attenuate muscle wasting through early recognition of the loss of muscle mass and effective personalized nutritional support during these therapies. Further studies are required to investigate TKIs to understand whether muscle mass could be a prognostic factor and whether early planning of nutritional strategies could improve prognosis.

The major strength of this systematic review lies in the gathering and analysis of the latest research on all TKI therapies, muscle mass, and clinical outcomes. Nevertheless, some weaknesses should be considered. First, for the majority of TKIs such as alectinib, bosutinib, cabozantinib, ceritinib, crizotinib, dasatinib, and nilotinib, no studies were found. Indeed, most data about toxicity and survival outcomes have focused on sorafenib. This bias could be explained by the fact that, in the setting of HCC and thyroid cancer, the real-life scientific research has mainly focused on sorafenib, since it was approved 12 years ago [55]. Secondly, data from the studies, retrospective in nature, are observational; therefore, the possibility of confounding and causal interference cannot be excluded. Randomized controlled trials (RCTs) could eliminate bias in treatment assignment, specifically selection bias, and confounding; however, RCTs evaluating the impact of a low mass in clinical outcomes without any support might be unethical. Another limitation is the high heterogeneity between studies because different sex-specific cut-offs were used, either based on study-specific medians [36, 42] or cut-off values from the literature [37, 38, 40, 50, 54]. Additionally, almost all of the studies did not have sufficient follow-up time for toxicity outcomes or reported completeness of follow-up, and included a limited number of patients, restricting the statistical power to detect an association. Finally, we only included English language studies thereby introducing language bias leading to potential publication bias. However, previous studies have failed to demonstrate a systematic bias from the use of language restriction [78, 79].

Notwithstanding these limitations, we can conclude that a low muscle mass during sorafenib therapy is significantly associated with worse outcomes in terms of OS and DLT, the last, especially in HCC patients. Moreover, the effect of muscle mass on clinical outcomes during all other TKI therapies remains almost unexplored. Studies with larger sample sizes, preferably using a prospective study design and with sufficient follow-up time, are needed to highlight the importance of the assessment of muscle mass as part of routine clinical practice for cancer patients undergoing TKI therapy. Information obtained from such studies could clarify the role of muscle mass in the metabolism of TKI-based cancer treatment, and its association with toxicity and survival. It could be crucial to establish, in clinical prospective studies or a real-world context, if an early muscle mass loss, induced by the disease itself but eventually worsening during TKI therapy, is not only a negative prognostic factor but also a potential surrogate for response, duration of response and overall survival.

## Supplementary Information

Below is the link to the electronic supplementary material.

Supplementary file1 (DOCX 506 KB)
