# Supplementary material for: Prognostic value of skeletal muscle mass during tyrosine kinase inhibitor (TKI) therapy in cancer patients: a systematic review and meta-analysis
Source: Intern Emerg Med. 2020 Dec 18;16(5):1341–56. doi: 10.1007/s11739-020-02589-5 (PMC8310498; doi:10.1007/s11739-020-02589-5)

**Supplementary file**

**Appendix 1:** Full search strategies for electronic databases

| **Database** | **PUBMED** | |
| --- | --- | --- |
| **Date** | **25 06 20** | |
| **Search strategy** | | **Results** |
| **#1** | Afatinib OR Alectinib OR Axitinib OR Bosutinib OR Brigatinib OR Cabozantinib OR Ceritinib OR Crizotinib OR Dasatinib OR Erlotinib OR Gefitinib OR Ibrutinib OR Imatinib OR Lapatinib OR Lenvatinib OR Nilotinib OR Osimertinib OR Pazopanib OR Ponatinib OR Regorafenib OR Ruxolitinib OR Sorafenib OR Sunitinib OR Vandetanib OR (tyrosine AND kinase AND inhibitor) OR TKI* | 99,135 |
| **#2** | "sarcopenia"[MeSH Terms] OR (muscle OR muscular*) OR (lean AND body AND mass) OR (body AND composition) OR (fat AND free AND mass) | 1,250,946 |
| **#3** | **#1 AND #2** | **4,007** |
| **Database** | **WEB OF SCIENCE** | |
| **Date** | **25 06 20** |  |
| **Search strategy** | | **Results** |
| **#1** | Afatinib OR Alectinib OR Axitinib OR Bosutinib OR Brigatinib OR Cabozantinib OR Ceritinib OR Crizotinib OR Dasatinib OR Erlotinib OR Gefitinib OR Ibrutinib OR Imatinib OR Lapatinib OR Lenvatinib OR Nilotinib OR Osimertinib OR Pazopanib OR Ponatinib OR Regorafenib OR Ruxolitinib OR Sorafenib OR Sunitinib OR Vandetanib OR (tyrosine kinase inhibitor) OR TKI | 131,621 |
| **#2** | ("sarcopenia"[Topic] OR (muscle OR muscular*) OR (lean AND body AND mass) OR (body AND composition) OR (fat AND free AND mass) | 1,062,536 |
| **#3** | **#1 AND #2** | **4,574** |
| **Database** | **SCOPUS** | |
| **Date** | **25 06 20** | |
| **Search strategy Results** | | |
| **#1** | Afatinib OR Alectinib OR Axitinib OR Bosutinib OR Brigatinib OR Cabozantinib OR Ceritinib OR Crizotinib OR Dasatinib OR Erlotinib OR Gefitinib OR Ibrutinib OR Imatinib OR Lapatinib OR Lenvatinib OR Nilotinib OR Osimertinib OR Pazopanib OR Ponatinib OR Regorafenib OR Ruxolitinib OR Sorafenib OR Sunitinib OR Vandetanib OR (tyrosine kinase inhibitor) OR TKI | 79,899 |
| **#2** | ("sarcopenia"[Topic] OR (muscle OR muscular*) OR (lean AND body AND mass) OR (body AND composition) OR (fat AND free AND mass) | 1,847,744 |
| **#3** | **#1 AND #2** | **5,204** |

**Appendix 2.** Assessment of the methodological quality of the eligible studies according Newcastle Ottawa Scale (NOS) criteria (+, yes; -, no; ?, unclear).


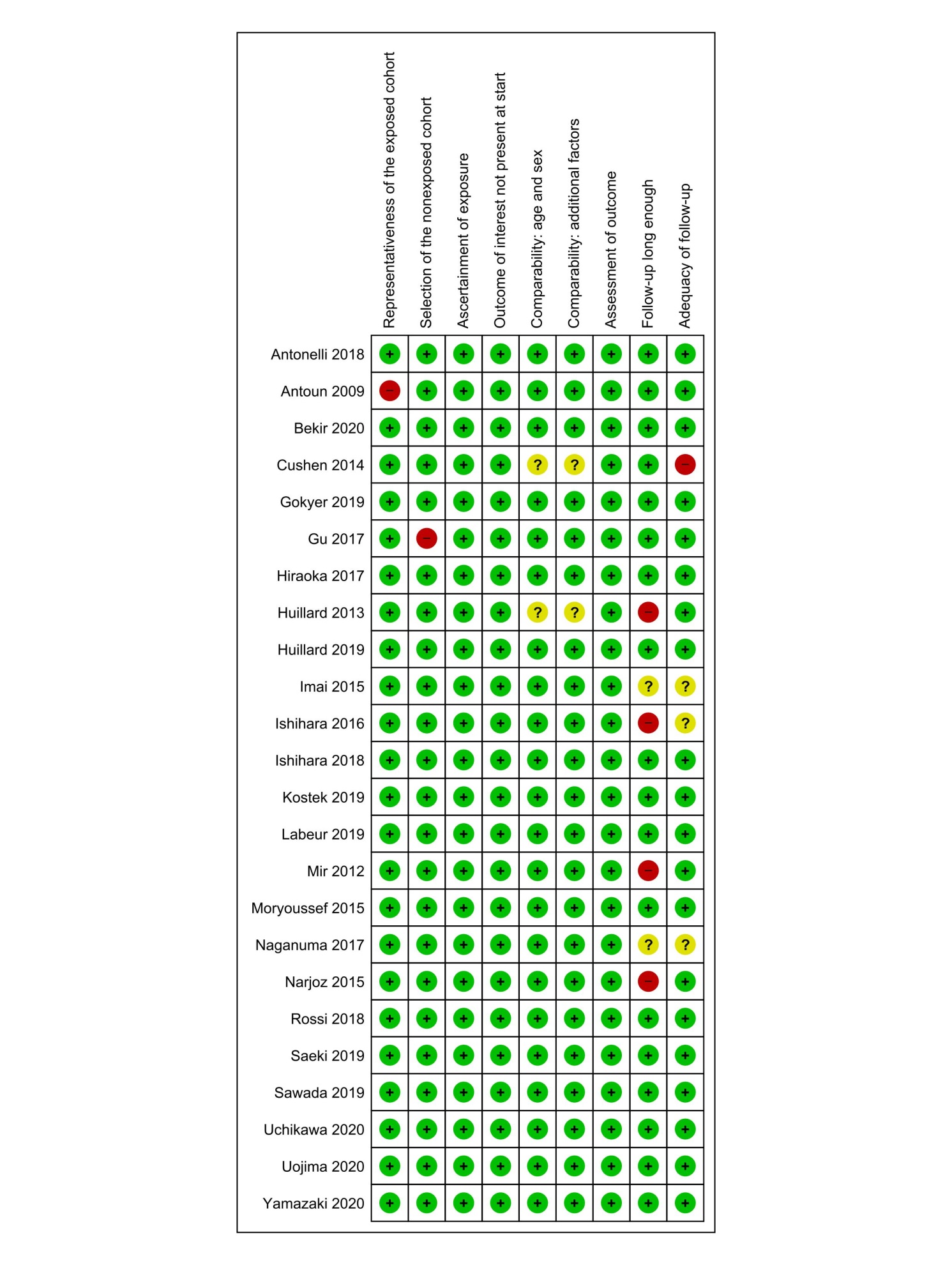

Supplement: Supplementary file 1 — Supplementary file1 (DOCX 506 KB) [file 11739_2020_2589_MOESM1_ESM.docx]
